# Latest advances in extracellular vesicles: from bench to bedside

**DOI:** 10.1080/14686996.2019.1629835

**Published:** 2019-06-17

**Authors:** Tomofumi Yamamoto, Nobuyoshi Kosaka, Takahiro Ochiya

**Affiliations:** a Division of Molecular and Cellular Medicine, National Cancer Center Research Institute, Tokyo, Japan; b Clinical Physiology and Therapeutics, Keio University Faculty of Pharmacy, Tokyo, Japan; c Department of Translational Research for Extracellular Vesicles, Tokyo Medical University, Tokyo, Japan; d Department of Molecular and Cellular Medicine, Institute of Medical Science, Tokyo Medical University, Tokyo, Japan

**Keywords:** Extracellular vesicles, exosomes, drug delivery system, biomarker, EV-based therapy, 30 Bio-inspired and biomedical materials, 600 Others

## Abstract

Extracellular vesicles (EVs) are small membraned vesicles and approximately 50–150 nm in diameter. Almost all of the type of cells releases the EVs and circulates in the body fluids. EVs contain multiple functional components, such as mRNAs, microRNAs (miRNAs), DNAs, and proteins, which can be transferred to the recipient cells, resulting in phenotypic changes. Recently, EV research has focused on their potential as a drug delivery vehicle and in targeted therapy against specific molecules. Moreover, some surface proteins are specific to particular diseases, and therefore, EVs also have promise as biomarkers. In this concise review, we summarize the latest research focused on EVs, which have the potential to become a promising drug delivery method, biomarker, and new therapeutic target for improving the outcomes of cancer patients.

## Graphical Abstract

**Figure:**
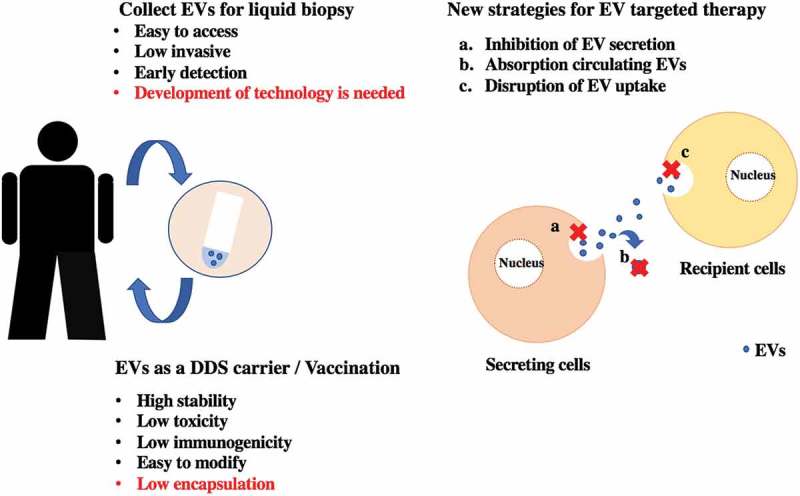

## 1. Introduction

**Figure 1.**
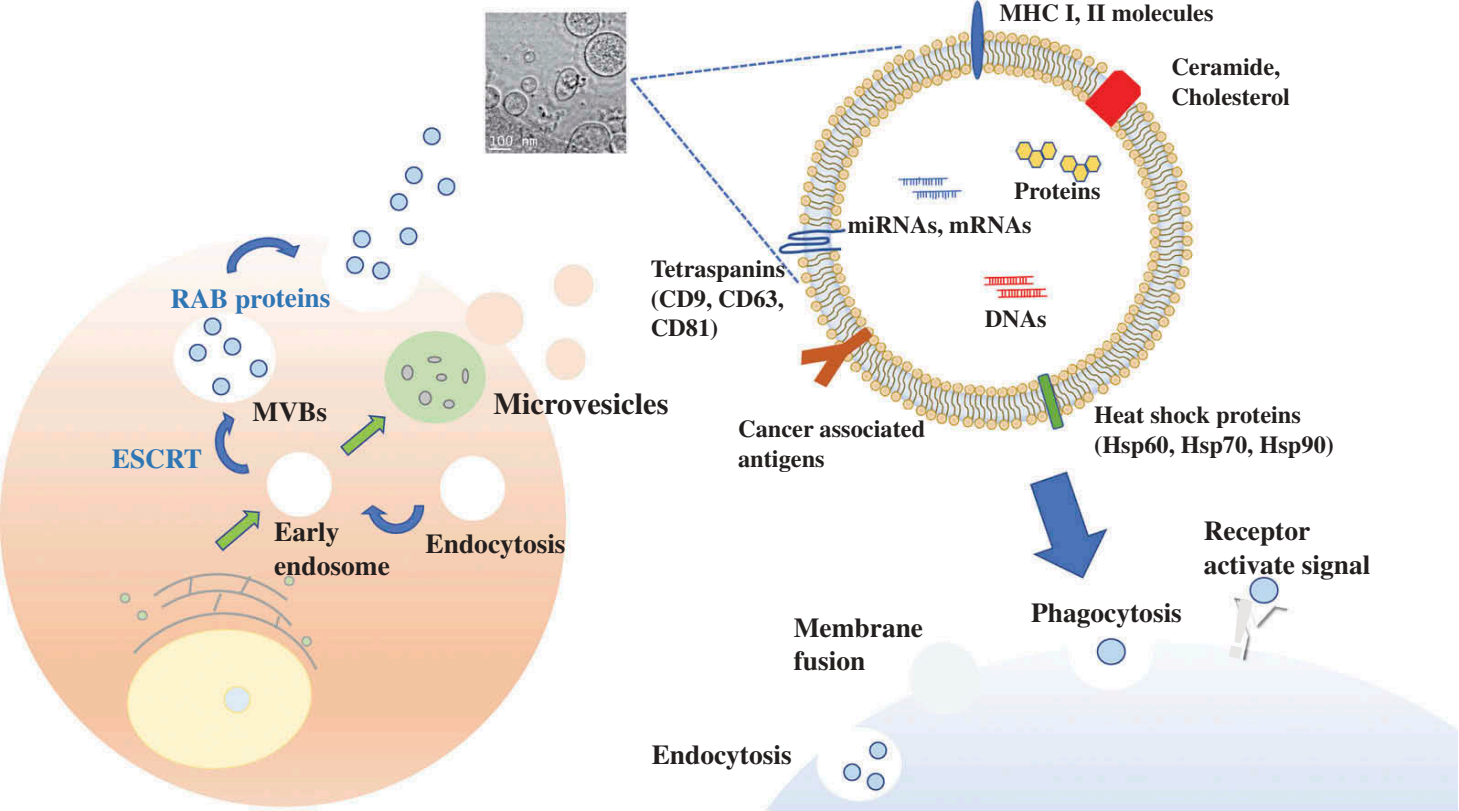
EV production procedure. Exosomes, 50–150 nm, are initially formed by a process of inward budding in early endosomes to form multivesicular bodies. Microvesicles are larger than exosomes, approximately 100–1000 nm. They are composed of lipid components and are directly shed or budded from plasma membranes. RAB proteins (RAB27A, RAB27B), ESCRT (Alix, TSG101) are associated with EV secretion. There are some markers on EV membranes, which is useful to detect EVs. EVs vary in size, properties, and secretion pathway depending on the originated cells, and the EVs are indeed taken up by recipient cells via a variety of mechanisms. Electron microscopy indicates the EVs derived from MCF7 breast cancer cell lines.

It has been shown that almost all of the cells release various types of extracellular vesicles (EVs), including exosomes, microvesicles, and apoptotic bodies. EVs vary in size, properties, and secretion pathway depending on the originated cells, and the EVs are indeed taken up by recipient cells via a variety of mechanisms (Figure 1) [1,2]. Exosomes are small EVs (sEVs), their diameter is approximately 100 nm. Exosomes are initially formed by a process of inward budding in early endosomes to form multivesicular bodies (MVBs) and released into the extracellular microenvironment to transfer their components [3,4]. Microvesicles (MVs) are larger than exosomes, approximately 100–1000 nm, and are composed of lipid components of plasma membrane [5]. MVs are synthesized in directly shedding or budding from plasma membranes. Apoptotic bodies have various sizes (1–5 μm), and only when cells are killed by the process of programmed cell death, resulting in secretion of apoptotic bodies. These various types of EVs have similar characteristics, such as size and density. Thus, more detailed classification is required for EV research. Although the role of EVs was initially supposed to be cellular waste management, such as, throwing unwanted proteins and biomolecules [6], in 2007, Valadi et al. have shown that EVs have contained mRNA in their lumen as well as microRNAs (miRNAs), which is considered a novel cell to cell communication tools [7]. In a few years from that year, several groups demonstrated that EVs transferred their functional miRNAs to recipient cells [8–11].

EVs from normal cells can affect their microenvironment. EVs derived from normal cells contribute to maintain homeostasis in their microenvironment, and it was shown that they can prevent cancer initiation [9,12]. For instance, Takahashi et al. demonstrated that the inhibition of EV secretion results in the accumulation of nuclear DNA in the cytoplasm, leading to the reactive oxygen species dependent DNA damage response, which induces the cell apoptosis [12]. On the other hands, tumor-derived EVs, including miRNAs, mRNAs, DNA, and proteins, also target various cell types to modify the tumor microenvironment and support the growth of tumor cells [11]. From these points of view, it has been shown that EVs are associated with physiological and pathological phenomena. With these advances, it is becoming possible to develop EV-based therapy.

In this review, we summarize the recent advances in EV research from bench to bedside. First, we discuss basic information about EVs. Second, we introduce the latest reports, which describe communication via EVs. Third, we focus on the current development of EV-based therapy, which has led to breakthroughs in therapeutics for cancer patients: 1, EVs as a vehicle for a drug delivery system (DDS); 2, EV components as diagnostic or prognostic biomarkers; and 3, antibody therapy against EV surface markers. Finally, we describe the issues remaining to be solved to allow EV-based therapy.

## 2. Basic information about EVs

EVs have been observed in all body fluids, such as blood, urine, saliva, sputum, breast milk, semen, and cerebrospinal fluid [13–19]. EVs have been shown to contain miRNAs, mRNA, DNAs, and proteins within the lipid bilayer [11]. This bilayer construction, which is very stable, enables EVs to circulate intact through body fluids, and thus, EVs can transfer their components to distant recipient cells. The accumulating data have indicated that the contents, size, and membrane composition of EVs are highly heterogeneous, dynamic and depend on the cellular source, state, and environmental conditions. EVs have some exosomal markers, including members of the tetraspanin family (CD9, CD63, CD81), members of the endosomal sorting complex required for transport (ESCRT) (TSG101, Alix), heat shock proteins (Hsp60, Hsp70, Hsp90), and RAB proteins (RAB27 A/B) [20,21]. Cells release heterogeneous vesicles of different sizes and intracellular origins, including small EVs formed inside endosomal compartments and EVs of various sizes budding from the plasma membrane [22]. Differential separation by immuno-isolation using either CD63, CD81, or CD9 was proposed. Several classically used exosome markers, like major histocompatibility complex (MHC), flotillin, and Hsp70 proteins, are similarly present in all EVs, and they also identified proteins specifically enriched in small EVs, and defined a set of five protein categories displaying different relative abundance in distinct EV populations. In this way, understanding the heterogeneity of EVs is important for EV-based therapy, since each EV has difference of their biological function on their membrane surface.

For a long time, only their functional abilities of EV are focused on, because the process from isolation step to detection step of EV is very complicated. To elucidate the biology of EV, highly purified EVs are essential for EV research. Typically, the ultracentrifugation (UC) method is used to collect EVs. Even today, the UC method is a general EV purification method; however, it has been reported that EVs purified by the UC method have low purity, the structure of the EVs is damaged by the high-gravity conditions, and the process is time consuming [23]. Thus, several other EV isolation methods have been developed, such as differential ultracentrifugation, density gradient centrifugation (sucrose or iodixanol gradients), filtration and size-exclusion chromatography, polymer-based precipitation, the antibody-coated immunobead method, ultrafiltration, and affinity chromatography [23–36]. Each method has advantages and disadvantages in cost, purity, and convenience. For instance, the advantages of the ultrafiltration method are simplicity, speed, and the ability to process many samples simultaneously; however, there are some disadvantages, such as protein contamination and poor biological activity [31]. We summarized the advantages and disadvantages of various EV purification methods in Table 1. Recently, new EV purification methods using microfluidic devices have been developed. Mengxi et al. have shown that a separation method based on acoustofluidics, the fusion of acoustics and microfluidics, can isolate EVs directly from whole blood in a label-free and contact-free manner [37]. This method uses two types of sequential surface acoustic wave microfluidic modules; one is a cell-removal module, and the other is an EV isolation module. These modules enable the isolation of EVs from undiluted blood samples with high purity and yield. As we have shown above, the development of EV isolation methods has been remarkable; however, there are still some issues of cost, convenience, and time consumption.

**Table 1.** Comparison of the advantages and disadvantages of various EV isolation methods.

Method	Advantage	Disadvantage	References
Ultracentrifugation & differential ultracentrifugation	Most common method, isolation from large sample volumes and multiple samples, no additional reagents	Time consuming procedure, EVs damaged from high speed centrifugation, contamination of non-EV fraction	[25,32–33]
Density gradient centrifugation (sucrose or iodixanol gradients)	High purity, no additional reagents	Complex procedure, loss of samples, affected by ultracentrifugation time	[28,35,37]
Size-exclusion chromatography	High purity and high reproducibility, reduced sample loss and EV aggregation, no additional reagents	Limitation on sample volumes, complex procedure, necessity of additional equipment, only one sample in each column, high cost	[27,34]
Polymer-based precipitation	Low cost, simple procedure,	Contamination and retention of the polymer	[31]
Antibody-coated immunobead method	High purity and high selectivity	Difficulty with detachment of the molecules, non-sopecific binding, existence of intact EVs, high cost	[26,29]
Ultrafiltration	Simple and fast procedure, the ability to process many samples simultaneously, no additional reagents	Loss of samples, contamination, poor biological activity	[36]
Microfluidic methods	High purity and efficiency,	Complexity of devices, necessity of additional equipment, high cost	[39,84,85]

## 3. Pathological roles of EVs

Over the last decade, accumulating evidence has shown that EVs can communicate between cancer and normal cells. In cancer, there are various phases, such as initiation, progression, metastasis, and recurrence. Tumor-derived EVs are closely associated with each phase and target various cell types to modify the tumor microenvironment to support the growth of tumor cells by inducing angiogenesis, tumor cell migration and metastasis, immune response modulation, and drug resistance [3]. Cancer cells are placed under low oxygen concentration, since there are few blood vessels carrying oxygen around tumor microenvironment, resulting in cancer cells become hypoxic. Hypoxia also leads to a change in the cargo within EVs, which induces angiogenesis in the nearby or distant microenvironment [38,39]. Hypoxia-inducible factor-1 promotes the production of angiogenesis-related genes, such as vascular endothelial growth factor. Abnormal angiogenesis in tumors is considered a major factor in cancer proliferation, therapy resistance, and metastasis. Treps et al. revealed that EVs are produced by glioblastoma multiforme (GBM), which contain a subpopulation of tumor cells with stem-like properties, termed glioblastoma stem-like cells (GSCs) [40]. VEGF-A is carried in EVs secreted from GBM patient-derived GSCs, and then, VEGF-A increases permeability and angiogenic potential in human brain endothelial cells. In the initiation phase of cancer, angiogenesis is very important for cancer survival. In the progression and metastasis phase, primary tumor cells can drive tumor progression through the transfer of EVs [41]. Zhen et al. demonstrated that miR-25-3p, a metastasis-promoting miRNA of colorectal cancer (CRC), can be transferred from CRC cells to endothelial cells via EVs, which consequently promotes vascular permeability and angiogenesis [42]. In addition, miR-25-3p from CRC cell derived EVs dramatically induces vascular leakiness and enhances CRC metastasis in liver and lung of mice. Tominaga et al. showed that miR-181c in EVs derived from breast cancer cells contributes to breaking the blood–brain barrier through the abnormal localization of actin via the downregulation of the miR-181c target gene, PDPK1, which leads to the downregulation of phosphorylated cofilin and the resultant activated cofilin-induced modulation of actin dynamics [43]. In another study, interactions of surface EV proteins with breast cancer cells lead to consequent activation of focal adhesion kinase signaling in the tumor cells, resulting in metastatic dissemination in a dose-dependent manner [44]. In addition to this, EVs are also related to long-term recurrence. For instance, breast cancer patients often develop recurrence, even more than 10 years after resection of their primary site, especially in the bone marrow [45–47]. Recurrence in the bone marrow indicates that cancer cells survive for a long time in a dormant state and migrate from the primary site to the bone marrow. Ono et al. demonstrated the mechanisms of the maintenance of dormancy in bone marrow via EVs derived from bone marrow mesenchymal stem cells (BM-MSCs), which transfer miR-23b to their target gene, myristoylated alanine-rich C-kinase substrate (MARCKS) [48]. Downregulation of MARCKS leads to inhibition of cell cycling and motility. EVs also acts as an oncogenic signal, which promotes an exit from dormancy. The horizontal transfer of mitochondrial DNA from EVs promoting an exit from dormancy of therapy-induced cancer stem-like cells and leading to endocrine therapy resistance in breast cancer [47]. These results indicate that EVs derived from cancer cells play a crucial role in cancer survival. Another example of induction of recurrence via EV, in glioma cells, glioma-astrocyte interaction plays an important role in tumor microenvironment remodeling. O6-alkylguanine DNA alkyltransferase (MGMT) mRNA in EVs released by reactive astrocyte is taken up by MGMT-negative glioma cells, which induce a temozolomide-resistant phenotype via the translation of MGMT mRNA in EV [49].

Of course, tumor derived EVs have ability to modulate immune systems to survive tumor cells. For instance, EVs can interact with recipient cells through ligand-receptor interactions. Specific EV proteins activate downstream pathways in recipient cells [50–52]. It has been shown that tumor cells evade immune surveillance by upregulating the surface expression of programmed death-ligand 1 (PD-L1), which interacts with programmed death-1 (PD-1) receptor on T cells to elicit the immune checkpoint response [53]. EVs derived from melanoma cells carried PD-1 on their surface, which suppressed the function of CD8 T cells.

**Figure 2.**
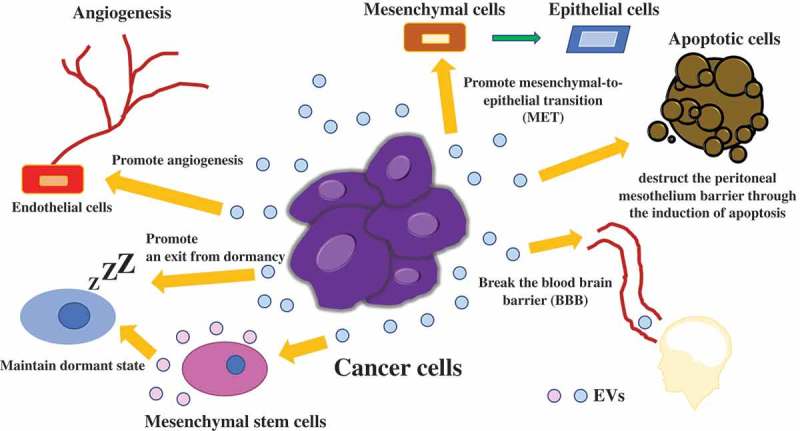
Communication in the tumor microenvironment via EVs. Intercellular communication via EVs contributes to various phases of tumor progression through the transfer of their components, such as, miRNAs, mRNAs, DNAs and proteins.

As we summarized above, intercellular communication via EVs contributes to various phases of tumor progression or suppression through the transfer of their components (Figure 2). These pathological roles of EVs strongly indicate that EVs are potent agents for cancer therapy. Although further research and development are needed for implementation before clinical use, we think EV-based therapy will be very powerful and promising for cancer patients. EV research is developing quite rapidly, and more detailed elucidation of the communication mechanisms between cancer cells and microenvironmental cells via EVs will enable cancer patients to be treated with EV-based therapy in the near future. From next session, we introduce a few examples of EV-based therapy.

## 4. EVs: potent agents for therapeutics

### 4.1. EVs as a vehicle for a drug delivery system (DDS)/EV-based vaccination

Cancer therapy based on anticancer drugs is used for many cancer patients after resection and sometimes in progressed patients whose tumors cannot be removed [54,55]. It is effective to some extent; however, there are issues that anticancer drug treatment always causes side effects, and the drug concentration is low in tumor sites [56]. It is difficult to accumulate the drug in the tumor sites, thus, it is impossible to avoid the influences on the surrounding normal cells. DDS, which can accumulate the drug at the tumor sites, has been focused. The advantages of DDS are enhancement of efficacy, reduction of side effects, improvement of safety, and reduction of medical expenses, which can lead to improvements in patient quality of life [57–60]. High binding capacity inhibits the drug from reaching the deepest parts of the tumor [61,62]. Recently, EVs as DDS agents have been investigated [63,64]. Ohno et al. demonstrated that EVs can efficiently deliver miRNA to the epidermal growth factor receptor (EGFR)-expressing breast cancer cells [65]. The GE11 peptide specifically binds to EGFR, which is less mitogenic than EGF, was over-expressed on the surface of EVs. These modified EVs could deliver let-7a in an EGFR-expression-dependent manner. These results suggested that EVs can be used therapeutically to target EGFR-expressing cancerous tissues with nucleic acid drugs. It has been shown that EVs can deliver a variety of bioactive cargos including small molecules [66–69], proteins [70], and siRNAs [71–73]. Srivastava et al. demonstrated that nanosized cellular vesicles, such as exosomes can transfer gold nanoparticle (GNP) therapeutic complexes without causing any particle aggregation or immune response [74]. This system consists of GNPs conjugated to the anticancer drug doxorubicin (Dox) by a pH-cleavable bond that is physically loaded onto the exosomes (Exo-GNP-Dox). The enhanced rate of drug release under acidic conditions led to successful uptake of this modified exosomes by the recipient cells. Another example of modified exosomes, exosomes fusing with liposomes by the freeze-thaw method was developed [75,76]. It was shown that this method optimized the properties of the exosome surface in order to decrease its immunogenicity and increase its colloidal stability, improving the half-life of exosomes in blood. In addition, the properties of the exosome surface could be modified using liposomes embedded with peptides or antibodies as targeting moieties or polyethylene glycol. Furthermore, it was shown that these hybrid exosomes efficiently encapsulated large plasmids, such as the CRISPR–Cas9 expression vectors [77]. Kamerkar et al. demonstrated that engineered exosome, called iExosome, which derived from normal fibroblast-like mesenchymal cells were engineered to carry siRNA or shRNA specific to oncogenic KRAS^G12D^ suppress pancreatic cancer [78]. In this study, Alexa-Fluor 647 (AF647) tagged siRNA was electroporated. Electroporation is one of the methods for siRNA encapsulation, and quantification of AF647-tagged RNAi containing exosomes isolated from the plasma of 24 hours post-injection was conducted. The signal of AF647-tagged siRNA was detected about 60% of EVs after 24 hours of injection. Compared with the electroporation treated siRNA alone without exosome, iExosomes with siRNA significantly suppressed pancreatic cancer and lead to the prolongation of overall survival in mice. Now, Clinical trials using iExosome are currently progressing to phase 2 [79]. There are many ongoing clinical trials using EVs (https://clinicaltrials.gov/ct2/results?cond=&term=exosome&cntry=&state=&city=&dist=), thus, in the near future, we believe therapeutic tools by EVs could be used for disease treatment.

As a drug delivery vehicle, bovine-milk-derived EVs are a topic of study. Bovine-milk-derived EVs are inexpensive, easy to collect in large amounts, and low toxicity [80,81]. From these points of view, a drug delivery system using bovine-milk-derived EVs is promising; however, the low encapsulation rate is the most significant issue. The methods of encapsulate molecules into EVs; To apply EVs as a DDS, it is important to include a ‘drug’ in the EVs (by attachment or encapsulation), preserve the EVs, and establish organ tropism. To the best of our knowledge, no suitable methods of preserving EVs as a DDS have been reported. Further development of EV research for using EVs as a DDS carrier is needed.

Not only as a carrier of DDS, EV is also attracting attention as vaccine. It is considered that EVs are powerful tools of cell free vaccination. Zitvogel et al. showed that tumor peptide-pulsed dendritic cell-derived EVs induced specific cytotoxic T lymphocytes activation and leading to suppress growth of established murine tumors in a T cell-dependent manner [82]. EVs could be expressed specific molecules of the cells from which they originate [83–87]. Inspired from the way that most enveloped viruses invade a host cell membrane and subsequently release by a budding process that requires cell membrane scission. Zhang et al. showed that they genetically engineered viral antigen to harbor into cell membrane, then form uniform spherical virus-mimetic nanovesicles (VMVs). These nanoparticles resemble EVs such as, in size, shape and specific immune function to elicit robust immunogenicity [84]. VMVs are considered to be straightforward, robust and tunable nanobiotechnology platforms for fabricating antigen delivery systems against a wide range of enveloped viruses, however, attention should be paid to the potential toxicity and biocompatibility of VMVs before this strategy may be translated into clinic.

### 4.2. EV components as diagnostic or prognostic biomarkers

**Figure 3.**
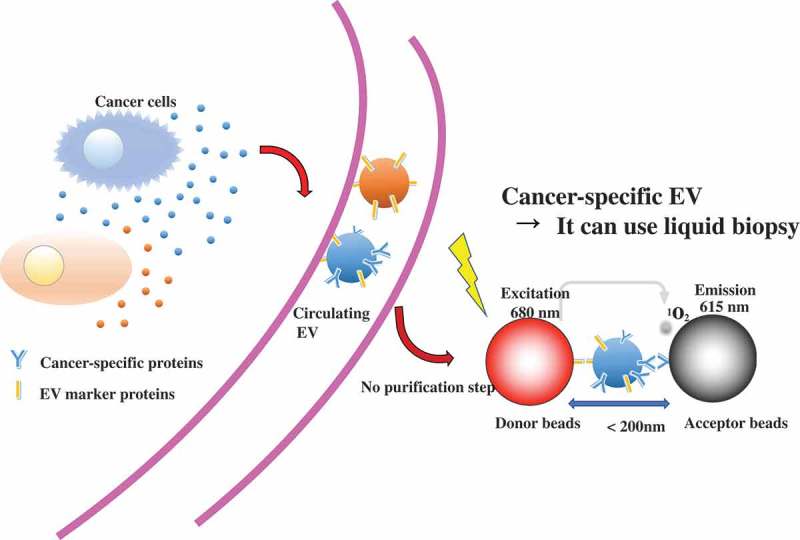
ExoScreen method is suitable for liquid biopsy. In this method, EVs are captured by two types of antibodies and detected by photosensitizer-beads, which enable the detection of EVs without sample purification in a small volume of body fluids or conditioned medium. The combination of antibody against EV specific marker and antibody against cancer-specific marker enable to detect the cancer-specific EV.

Currently, a biopsy is required for definite diagnosis of many cancers. Biopsy imposes a heavy burden on patients, and because of this burden, it is difficult to perform biopsies many times. It is necessary to observe the cancer phases and the response to treatment. Thus, rapid, simple, and less invasive diagnostic methods are needed. Liquid biopsy is a predictive diagnosis and treatment method using body fluid samples, such as blood [88,89]. EV-based liquid biopsy has some advantages compared with conventional biopsy. One of them is that, because EVs are secreted from almost all cells, EVs are found in various body fluids, and easy to collect [90]. Second, EVs have biomolecules on their surface that might represent the status of the disease. In addition to this, miRNAs in the serum EVs from cancer cells have been observed [91]; therefore, the miRNAs, as well as proteins, in EVs are expected to serve new biomarkers. A recent study showed another component of EVs, mRNA, also has the potential to provide biomarkers for cancers. Yokoi et al. revealed that MMP1 mRNA in ovarian-cancer-derived EVs is carried to the peritoneal cavity through the ascites, and then, induction of apoptosis in the mesothelial cells leads to the destruction of the peritoneal mesothelium barrier [92]. In this report, it was shown that the expression level of MMP1 mRNA from ovarian cancer patient ascites is higher than that in healthy donors. This result indicates that MMP1 mRNA in ascitic EVs has potential utility as a risk indicator of peritoneal metastasis. Furthermore, to evaluate mRNA level in EVs, a configurable microwell-patterned microfluidic digital bioassay chip was developed. This device enables to detect absolute quantification of mRNAs in tumor derived EVs with high sensitivity and specificity [93]. Another example of liquid biopsy, androgen receptor splice variant 7 mRNA expression in urinary EVs can be reliably quantified in prostate cancer patients [94] Absolute quantification using droplet digital polymerase chain reaction provided sensitive detection of urinary mRNA from intact EVs, which were prepared by label-free, size-based enrichment using a centrifugal microfluidic device. Furthermore, tumor derived EVs contain circulating orphan noncoding RNAs (oncRNAs), which may also have a role in non-cell autonomous disease pathogenesis [95]. These oncRNAs exerts its prometastatic effects by acting as an inhibitor of RISC complex activity and increasing the expression of the prometastatic genes, NUPR1 and PANX2. As we have mentioned above, EV-based liquid biopsy is more convenient than conventional tissue biopsy; however, as a clinical technique, EV detection remains time consuming, and some additional criteria must be met, including cost, high sensitivity, high specificity, and correlation with the clinical process of the disease. A recent study showed that EV protein biomarkers on alternating current electrokinetic chips enable rapid detection of pancreatic cancer in patient blood [96]. This method enables users to distinguish pancreatic cancer patient samples with high sensitivity (99%) and specificity (82%) within two hours, and there is no need for any purification. There are various methods to detect EVs, such as using EV surface-anchored nucleic acid amplification [97], a label-free electrochemical sensor [98,99], and others [100]. To apply EV-based liquid biopsy, more improvement in earlier detection is important for successful treatment. Recently, we have established ExoScreen, which is a new, ultrasensitive EV detection method [101]. In this method, EVs are captured by two types of antibodies and detected by photosensitizer-beads, which enable the detection of EVs without sample purification in a small volume of body fluids or conditioned medium. The combination of antibody against EV specific marker and antibody against cancer-specific marker enables detection of the cancer-specific EV (Figure 3).

The development of new EV detection methods enables EV detection from small amounts of sample, such as clinical samples. In fact, CD147, which is considered a biomarker for early-stage colorectal cancer, and CD9 double-positive EVs were also detected in samples with early-stage colorectal cancer that had invaded the submucosal layer by the ExoScreen assay [101]. In this respect, the development of EV detection is also important for EV-based therapy. Recently, circulating RNAs in EVs are utilized for cancer diagnosis. Sedlarikova et al. demonstrated that two-phase biomarker study for lncRNA expression profiles in EVs from peripheral blood serum of newly diagnosed multiple myeloma (MM) patients, monoclonal gammopathy of undetermined significance (MGUS) patients or healthy donors were investigated [102]. Overall, MM and MGUS patients showed high expression of lncRNA, called PRINS. These results suggest that lncRNA, PRINS, might be a minimally invasive marker of both MM and MGUS.

### 4.3. Strategy of EV-targeting therapy

As we have shown, intercellular communication via EVs contributes to tumor progression through the transfer of their cargo. Another important issue is whether EVs from cancer cells can be therapeutic targets. EVs can progress cancer through pathways completely different from the molecules targeted by current drugs. Thus, reduction of cancer-derived EV cargo transmission may provide possibilities for new therapeutics for cancer patients and add additional value to existing therapeutic methods. We have proposed three potential EV-based therapeutic strategies; inhibition of EV production, elimination of circulating EVs, and disruption of the absorption of EVs. Elimination of circulating EVs is a possible new therapeutic strategy in cancer patients. For instance, antibodies against CD9 and CD63, which are enriched on EVs, were administered to human breast cancer xenograft mouse models, and circulating administered EVs tagged by anti-CD9 and -CD63 were internalized by macrophages through phagocytosis, resulting in the inhibition of cancer progression [103]. However, these CD9 or CD63 antibodies cannot selectively bind cancer-derived EVs; thus, further investigations to find cancer-specific antigens on the surface of EVs are needed. Indeed, another example strategy for eliminating EV using a cancer-specific antigen has been reported [104]. This study used the cancer-specific EV surface protein HER2. Because of this, the therapeutic strategy for the removal of circulating EVs was successful. The researchers developed the hemofiltration system, which can specifically capture circulating cancer-cell-derived HER2-positive EVs [104]. HER2-expressing EVs have been shown to interfere with therapy and are associated with cancer progression [105]; therefore, selectively eliminating HER-2-expressing EVs could be a new strategy to treat breast cancer.

Several articles have described the effectiveness of inhibiting EV production, which inhibits cancer progression *in vitro* and *in vivo*. Kosaka et al. revealed that knockdown of nSMase2, which is required for the synthesis of ceramide, reduced EV secretion and miR-210 transfer, resulting in suppression of angiogenesis and metastasis in a xenograft mouse model [106]. The lysosome-associated membrane protein-2 (LAMP-2) is associated with endocytosis [107], knockdown of LAMP-2 improved the therapeutic effect of sunitinib in pancreatic neuroendocrine tumors [108]. In addition, it has been shown that other molecules, such as RAB27A/B, TSG101, TSAP6, which are related to EV secretion, are involved in the EV secretion from cancer cells. Although therapeutic strategies for targeting EVs derived from cancer cells are effective, there are several issues to apply before clinical use. These genes have key roles in multiple cell biological phenomena, thus, their downregulation in normal cells would have deleterious effects on normal cell functions. Therefore, identification of the genes related to cancer-specific EV secretion mechanisms is needed before using EV-targeting therapy. It has also been suggested that EV-targeting therapy may affect normal cells [103]. To identify ‘cancer-specific molecules’ is indispensable for the future development of EV-targeting anticancer therapeutics. EV biogenesis is still unclear, and some challenging issues remain; however, the EV research field is developing rapidly. Thus, we hope that EV-targeting therapy will become a standard therapy for cancer patients.

## 5. Conclusions

**Figure 4.**
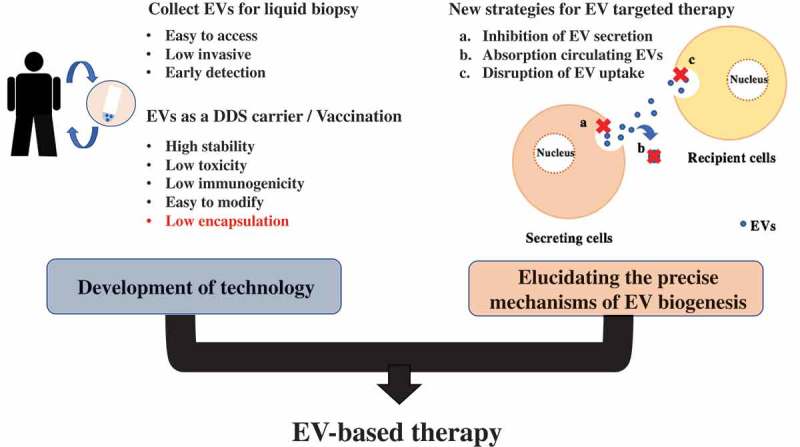
EVs are a powerful and promising tool for cancer therapy. EVs have potential as biomarkers, drug delivery vehicles, and new targets in cancer therapy. To use EVs for liquid biopsy, an easier and lower-cost method is needed. Low encapsulation ratio is the most significant issue for the use of EVs as a DDS carrier. There are still some issues to be solved to start EV-based therapy. EV-targeting therapy is very promising, because the pathways promoted by EVs are completely different from those targeted by existing anticancer drugs; however, the elucidation of cancer-specific EV secretory pathways or cancer-specific antigens on EVs is needed.

As we have mentioned above, EVs are a very powerful and promising tool for cancer therapy (Figure 4). As medical technology advances, the aging society is rapidly spreading all over the world, and the possibility of suffering from cancer is increasing remarkably. To treat cancer efficiency, early detection is the most important factor. As a biomarker, EVs are suitable for their feature of encapsulation of various molecules, which are specific for cancer types, and EV-based liquid biopsy has many advantages. Liquid biopsy is easy to access compared with conventional tissue biopsy. At the same time, these cancer-specific molecules can provide new therapeutic targets for cancer. The pathways associated with cancer progression via EVs are different from the targets of modern drugs. Additional efficacy is expected when these pathways are inhibited. Moreover, EVs are possible drug delivery vehicles, which can efficiently deliver various bioactive agents to target cells. Although advances in EV purification methods are developing remarkably, some issues remain to be solved before future clinical use will be possible.

For instance, the precise mechanisms of EV absorption pathway have not been clearly understood. Thus, for more efficient delivery, elucidating the precise mechanism of EV absorptive pathways are desired. In addition, the clinical use of EV-targeting therapy has also an issue to be solved. Since most types of cells secrete EVs through different pathways, EV-targeting therapy itself may affect the normal physiological function of EVs secreted from normal cells. Discovery of cancer-specific EV secretion pathway and/or proteins can dramatically improve EV-targeting therapy.

EV-targeting therapy has been significantly advanced recently. Many companies developed the technology of engineering EVs. Codiak® is one of the companies established a methodology of EV engineering. exoSTING® is composed of engineered EVs loaded with a small molecule STING agonist. The engineering of exoSTING® provides for selective, preferential STING delivery to antigen presenting cells [109,110]. A clinical trial using this modified EVs is ongoing. There is a need for a technology for purifying EVs stably and in large quantities; however, we believe that, in the near future, EV-based treatments will be used for cancer patients, and EV-based therapy will help to overcome cancer.
